# Some Scientific Aspects of Insanity

**Published:** 1892-03-05

**Authors:** 


### Some Scientific Aspects of Insanity.

XV.—SECONDARY DEMENTIA (*Continued*).

Paralytic dementia is that form of insanity which is associated with some extensive gross injury to the brain, which, in the great majority of cases, causes paralysis of some part of the body. The primary lesion is usually an apoplexy, which means the bursting of an artery into the brain substance, or softening of a portion of the brain, which is frequently caused in weak, elderly people by the blocking of one of the small cerebral arteries, and the consequent starvation and softening of that part of the brain which is supplied by the diseased artery.

The chief mental symptoms of paralytic insanity are:—

(*a*) Mental weakness. This symptom is the prominent and ever-present one in all such cases.

(*b*) There is also invariably present a greater or less degree of emotionalism, which tends to manifest itself on the very slightest occasion, or perhaps for no cause whatever.

(*c*) A feeble, ineffectual irritability of temper, generally manifested by misdirected explosions of anger, is also commonly met with in these cases.

(*d*) In a large proportion of patients thus afflicted there is a tendency to be noisy and restless, and sleepless during the night. It is this last symptom which makes most of the cases so distressing to their friends, and compels their removal to special institutions for the care of the insane.

All cases of paralytic insanity are not necessarily aged. Some of them are comparatively young, but it is often extremely difficult to distinguish between paralytic cases of insanity and the next group to be described, because many old people are feeble, and semi-paralytic, on account of a uniform degenerative process taking place in the elements of their nervous systems.

Senile dementia is a more or less natural process of mental decay, in so far as it necessarily occurs in healthy individuals, as the result of extreme old age. There is, however, a morbid form of senile dementia, the result of decay and degeneration of the tissues of the nervous system, which may occur at any age, but usually after sixty-five. The whole colour of the insanity is that of physical and mental weakness. There is a restlessness, which is characterised by a want of activity and of purpose. The voice is feeble and shaky, the movements of the muscles are weak. There is a failure of memory which differs from that we have described in alcoholic dementia, in that it is more general. The patient forgets words, faces, and surroundings, and seems neither to live in the present nor in the past. Although many cases begin with mania, and throughout the disease are subject to recurring attacks of maniacal excitement, the prevailing mental state is one of melancholia and depressed emotionalism. Hallucinations, delusions, and suspicions are common in many cases, and an irritability of temper similar to that which occurs in paralytic insanity is a frequent accompaniment of the malady. Sleeplessness, restlessness, and noise during the night are in a large proportion of cases the difficult and distressing symptoms, and when present demand, as in paralytic insanity, treatment in an asylum.

With regard to the treatment of secondary dementia, it may be generally stated that the first two forms (secondary dementia proper, and alcoholic dementia) do not, in the absence of complications, demand any special attention or nursing. That general careful supervision which is requisite for the welfare of the chronic insane is of primary importance. The more the cases are separated, individualised, and specially observed, the higher will be the standard attained in the morale and welfare of a chronic ward composed of such patients. In addition, the nurses and attendants require to judiciously assist in the employment and amusement of such cases.

For the feebler classes of secondary dementia, which include paralytic insanity and senile dementia, a much greater call is made upon the nursing instincts of the attendants. Above all, they require attention, constant supervision, and kindness. Nursing in its most literal sense, as well as in the highest sense, is necessary for their well-being. The habits of such patients are most frequently faulty and untidy, and as they have no capacity for training, the task of attention to these habits becomes an arduous and constant one. They require to be warmly clad, to be kept in an equable temperature, and lastly, and, chiefly, they require frequent supplies of food, and, where it is permitted by the regulations of the institution, a little stimulant is often necessary for their comfort. As they frequently complain of hunger between meals, it is proper and humane to appease their craving by such slight nourishment as a piece of bread and a little milk.

There is nothing that places the humane tendency of the age in such a good light as to enter the infirmary wards of a well-conducted modern asylum and to observe the care, the nursing skill, the cleanliness, and the comfort with which numbers of hopelessly enfeebled old women are surrounded. It has appeared to many to be ridiculous that so much labour and money should be expended upon prolonging the existence of worse than useless human life. Apart from higher arguments, which might be brought forward in abundance, it appears to me that if it is not our duty to do all in our power to alleviate the condition of these helpless beings and to prolong their life, then it should follow as a logical conclusion that we should endeavour to discover some painless method of destroying more than fifty per cent. of those who are annually committed to asylums for the insane. If such an idea is revolting and horrible, then it follows that we cannot do too much for suffering humanity, however enfeebled and worthless it may appear.

